# Revealing brain’s cognitive process deeply: a study of the consistent EEG patterns of audio-visual perceptual holistic

**DOI:** 10.3389/fnhum.2024.1377233

**Published:** 2024-03-27

**Authors:** Hongwei Li, Haifeng Li, Lin Ma, Diokova Polina

**Affiliations:** Faculty of Computing, Harbin Institute of Technology, Harbin, China

**Keywords:** information processing, event-related potentials, information-related potentials, informational attribute, perceptual holistic

## Abstract

**Introduction:**

To investigate the brain’s cognitive process and perceptual holistic, we have developed a novel method that focuses on the informational attributes of stimuli.

**Methods:**

We recorded EEG signals during visual and auditory perceptual cognition experiments and conducted ERP analyses to observe specific positive and negative components occurring after 400ms during both visual and auditory perceptual processes. These ERP components represent the brain’s perceptual holistic processing activities, which we have named Information-Related Potentials (IRPs). We combined IRPs with machine learning methods to decode cognitive processes in the brain.

**Results:**

Our experimental results indicate that IRPs can better characterize information processing, particularly perceptual holism. Additionally, we conducted a brain network analysis and found that visual and auditory perceptual holistic processing share consistent neural pathways.

**Discussion:**

Our efforts not only demonstrate the specificity, significance, and reliability of IRPs but also reveal their great potential for future brain mechanism research and BCI applications.

## 1 Introduction

Recorded at the human scalp through electrodes, electroencephalogram (EEG) reflects the spontaneous and rhythmic electrical activity of a population of brain cells. Ever since Hans Berger reported his discovery, EEG has been widely used to explore the cognitive processing mechanisms in the human brain (Jeong et al., 2022; Proix et al., 2022; Tamano et al., 2022). In Pauline and Davis et al. (1939) first observed and recorded stimulus-related evoked potentials on awake human brains (Davis et al., 1939). In Vaughan (1969) extracted evoked potentials from the EEG by averaging technique and proposed the concept of event-related potentials (ERPs). ERPs reflect the brain’s cognitive processes and are regarded as a window for observing mental activities (Kropotov, 2016). During more than a half-century of research, brain activity laws and patterns are investigated through all kinds of visual and auditory experiments (Hajcak et al., 2019; Eftimov et al., 2020; Grasby et al., 2020).

Contemporary research in cognitive science has demonstrated that the brain employs a hierarchical information processing principle when processing stimuli. Researchers have identified three stages of the brain’s cognitive process: the elementary stage, the intermediate stage, and the advanced stage (Van Essen et al., 1992; Blesa et al., 2021; Zhang et al., 2022).

During the elementary stage, the brain processes the fundamental physical attributes of stimuli, such as the frequency, intensity, and timbre of sounds, as well as the brightness, saturation, and orientation of images. In the study of cognitive science, this stage is called the sensory processing stage. In the field of artificial intelligence, this stage corresponds to the processing of physical attributes of samples or low-dimensional feature extraction. Throughout this article, we refer to this stage uniformly as the physical processing stage. ERP components (the visual C1, P1, and N1, the auditory N1, etc.) are all observed at this stage. Luck (2014) demonstrated that visual C1 is sensitive to the contrast and the spatial frequency of images. Varghese et al. (2017) reported the visual C1, P1, and N1 are influenced by perturbation characteristics, postural set and environmental factors . Rosburg and Mager (2021) indicated the amplitude of the auditory evoked N1 depends on the interstimulus interval (ISI). Thomson et al.’s (2009) experiments suggested that N1 reflects an auditory detector sensitive to the changes in rising time of sounds, the faster the sound reaches the highest amplitude, the higher the N1 intensity is observed. Since the physical attributes of stimuli are mainly processed in this stage, it is called the physical attribute processing stage in this article.

The intermediate stage focuses on how the brain processes image composition, texture and outline, sound melody, beat and rhythm, as well as other structural attributes of visual and auditory stimuli. This stage is the end of sensory processing and the beginning of perceptual processing. In this article, we refer to this stage as the structural processing stage. ERP components such as the visual N2, the auditory N2 and P300 are all observed at this stage. Courchesne’s work found that visual N2 correlated with stimulus structure. It was found that complex stimuli (i.e., complex, colorful patterns) elicited larger N2 amplitudes than simple stimuli (e.g., geometric figures) (Courchesne et al., 1975; Ragazzoni et al., 2019). Breton et al. (1988) demonstrated that changes in physical attributes such as contrast and brightness of the target stimulus did not cause changes in parietal P3b and temporo-occipital N2. A binaural split-listening experiment was used by Ozdamar et al. (2011). They found that the auditory N2 component correlated with the beat (Ozdamar et al., 2011). Celsis et al. (1999) found that the N2/P3 component highly correlated with speech processing. Kushnerenko et al. (2001) found that the N2 component correlated and increased with sound duration. All these discoveries indicate that during this stage the brain mainly processes the elements of stimuli. Therefore, we called this stage the elemental processing stage.

During the advanced stage, the brain focuses on abstract object recognition, semantic processing, and content comprehension. This stage represents the integration of the elementary and intermediate stages, specifically perceptual holistic or perceptual integration. These imply that the brain processes and comprehends the high-level attribute of stimuli during this stage. The high-level attribute of stimuli is the product of the interconnection and interaction of the physical and structural attributes in the stimuli. According to Shannon’s information theory, Information reflects the state of motion and change of various things in the objective world, a representation of the interconnection and interaction between objective things, and an expression of the substance of the state of motion and change of objective things. So, we refer to these high-level attributes of stimuli as information attributes of stimuli. Hence, we called this stage the informational processing stage. Current researches about informational stage focus on semantic processing, and the representative ERP component is N400. Kutas and Hillyard (1980) first reported this component in 1980 and demonstrated that N400 is highly correlated with language processing. Hodapp and Rabovsky (2021) found the interpretation of the N400 as an implicit learning signal driving adaptation in language processing. The N400 ERP was suggested as an important tool to assess information-processing capacities by Steppacher et al. (2013).

Besides these investigations, research on abstract object recognition and content understanding is rarely found, and corresponding experimental paradigms are missing. Therefore, based on the current ERP research on physical and elemental processing, this article proposed a novel method that exploits informational attributes of stimuli to study the brain information processing mechanism. The stimuli consist of several fixed elements, and the arrangement of elements reflects the informational attributes. Both visual and auditory information-related cognition experiments based on the Go-Nogo paradigm were conducted. Behavioral data and EEG data were recorded simultaneously. The attributes and parameters remained consistent throughout the experiment, except for the informational attributes. Consequently, the source of behavioral and EEG differences among subjects can be attributed solely to the informational attributes. By averaging and subtracting, we can obtain the evoked potentials induced by these informational attributes. We are committed to discover the brain’s unified EEG/ERP representation during the informational processing stage.

The data analysis includes four parts: (1) Analysis of behavioral data verified information processing stage existed in the experiment. (2) A series of IRP components were identified through the in-depth analysis of potential changes. (3) The Common Spatial-Spectral Pattern (CSSP) was used to identify IRP on single-trial EEG, further decoding the brain cognitive processing process. (4) Constructing brain networks for information processing and discovering similar neural pathways for visual and auditory information processing, further demonstrating the consistent EEG patterns of audio-visual perceptual holistic.

## 2 Materials and methods

### 2.1 Experimental procedure

In order to exploit the brain’s information processing mechanism, we conducted 2 cognition experiments that based on the Go-Nogo paradigm: the Visual Information Cognition Experiment (VICE) and the Auditory Information Cognitive Experiment (AICE). In the experiments, a variation of the Go/Nogo paradigm was employed, in which subjects were required to respond to both stimuli to ensure consistency in experimental conditions such as the presentation probability and the experimental task. By carefully designing two stimuli that differed only in information attributes, we ensured that any behavioral variances among participants and discrepancies in EEG during the experiment could only arise from alterations in information properties.

#### 2.1.1 Ethics statement

All experimental protocols were approved by Heilongjiang Provincial Hospital Ethics Committee. Informed consent was obtained from all subjects and/or their legal guardian(s). All procedures involving human participants were performed according to the ethical standards as laid down in the 1964 Declaration of Helsinki and its later amendments or comparable ethical standards.

#### 2.1.2 Stimulus

The core of our research is to define stimuli with the same physical and elemental attributes, but different informational attributes. In VICE, stimuli consist of the same number of elements. The specific arrangement of elements can form significant images (positive polygons). At this time, the brain not only processes physical and element attributes but also performs abstract object recognition, which is the information processing stage. In this article, such stimuli were used as Go stimuli. Conversely, the elements arranged randomly form meaningless images. Such stimuli were used as Nogo stimuli. The specific scheme is as follows.

**FIGURE 1:**
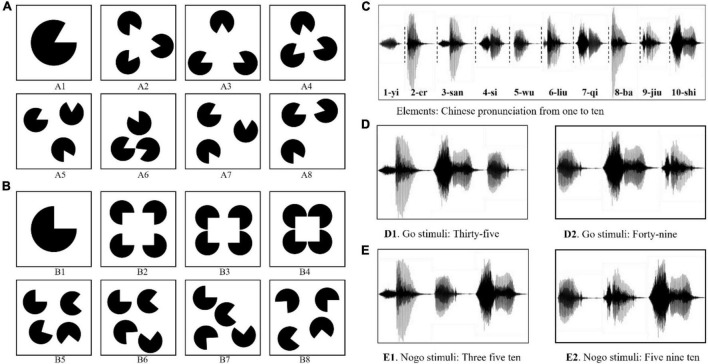
Examples of the stimulus materials in Information Cognition Experiment. **(A)** Panel A1 is the element in VICE. Panels A1–A4 are the examples of the Go stimuli. Panels A5–A8 are the examples of the Nogo stimuli. **(B)** Panel B1 is another element in VICE. Panels B1–B4 are the examples of the Go stimuli. Panels B5–B8 are the examples of the Nogo stimuli. **(C)** The elements used in AICE: the Chinese pronunciations of the 1∼10 digits. **(D)** Examples of the Go stimulus: the correct Chinese pronunciation of 35 and 48. **(E)** Examples of the Nogo stimulus: stimuli with no numerical meaning.

The visual stimuli were divided into two types: (1) The sector with a 300° circular angle was used as the element, as shown in Figure 1A1. Each stimulus contained three elements with identical size and shape. (2) The sector with a 270° circular angle was used as the element, as shown in Figure 1B1. Each stimulus contains four elements with identical size and shape. In Go stimuli, the elements were arranged in a specific arrangement to form information (e.g., positive triangles or positive quadrilaterals, as shown in Figures 1A2–4, B2–4). The elements in Nogo stimuli were randomly arranged and cannot form information, as shown in Figures 1A5–8, B5–8.

To further deepen our research on IRPs, we also conducted an Auditory Information Cognitive Experiment (AICE). Each auditory stimulus consisted of three elements. The elements of AICE were the Chinese pronunciations of the 1∼10 digits (/yi/ for 1, /er/ for 2, 3-/san/, 4-/si/, 5-/wu/, 6-/liu/, 7-/qi/, 8-/ba/, 9-/jiu/, and 10-/shi/, as shown in Figure 1C). When the alignment of elements conformed with Chinese grammar (expressing a two-digit number from 21 to 99 in Chinese), the brain would understand the information in the stimulus. Such stimuli were used as Go stimuli. Nogo stimuli were those in which the elements were randomly arranged and violated the grammar. The specific construction rules are as follows.

In the Go stimulus, /shi/ only appeared in the middle, such as /san shi wu/ and /si shi ba/, as shown in Figure 1D. They were the correct Chinese pronunciation of 35 and 48. The brain could understand its meaning normally. Thus, we considered them as stimuli with information.

In the Nogo stimulus, /shi/ appeared only at the end, such as /wu ba shi/, /wu san shi/, /si ba shi/, and so on. These stimuli violated Chinese grammar and had no numerical meaning. Hence, they were regarded as Nogo stimuli, as shown in Figure 1E.

The digital pronunciation was synthesized by professional software and unified in terms of duration and loudness. The element duration was 0.5 ms and the stimulus duration is 1.5 s.

#### 2.1.3 Participants

Twenty right-hand dominant, healthy Chinese speakers [age 21.5 ± 1.3 years (mean ± SD); 5 female] participated in the Visual Informational Cognitive Experiment. Nineteen right-hand dominant, healthy Chinese speakers [age 21.75 ± 1.8 years (mean ± SD); 3 female] participated in the Auditory Informational Cognitive Experiment. Seven subjects took part in both cognitive experiments. All participants are undergraduate students from the Harbin Institute of Technology. Their first foreign language is English. No participants reported hearing loss or a history of neurological illnesses. All participants had normal hearing and vision and were musical laymen with no professional musical education. They reported normal nocturnal sleep patterns (7–9 h starting from 10 p.m. to 12 a.m.) for the week before the experiment. They had not used caffeine, nicotine, or energy drinks and had not performed excessive exercise 24 h before the experiment.

#### 2.1.4 Equipment and procedure

**FIGURE 2:**
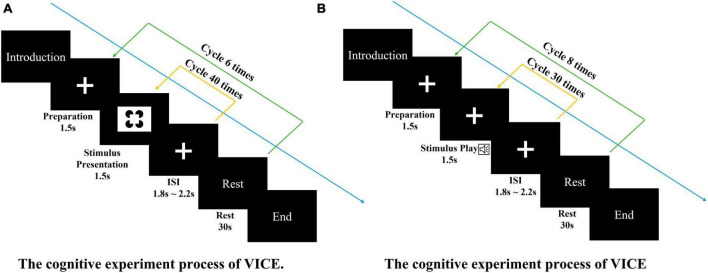
The cognitive experiment processes. **(A)** The cognitive experiment process of VICE. The visual task consisted of 6 blocks of 40 trials each (240 total trials). Each trial consisting of the following steps: (1) A 1.5-s baseline recording (fixation cross). (2) The 1.5-s display of the stimuli. (3) A short rest. Each block contains 30-s rest. **(B)** The cognitive experiment process of AICE. The auditory task consisted of 8 blocks of 30 trials each (240 total trials). Each trial consisting of the following steps: (1) A 1.5-s baseline recording (fixation cross). (2) The 1.5-s display of the stimuli. (3) A short rest. Each block contains 30-s rest.

Both cognitive experiments used the Go-Nogo experimental paradigm, in which Go stimuli or Nogo stimuli were randomly presented in a single sensory channel in a 1:1 ratio. Participants’ behavioral data and EEG data were collected. The participants were required to press one button for Go stimuli and another button for Nogo stimuli. The experimental process is shown in Figure 2.

The experimental design employed in this study is a modification of the Go-Nogo paradigm, wherein participants were tasked with responding to both types of stimuli through button presses. The rationale for employing this variant lies in two primary advantages: Firstly, it ensures complete consistency in the presentation of both types of stimuli throughout the experiment, differing only in their informational attributes. Secondly, it proves advantageous in acquiring behavioral data pertaining to participants’ processing of the two stimulus types. This enables a comprehensive analysis of participants’ cognitive processing from the vantage point of behavioral data.

**FIGURE 3:**
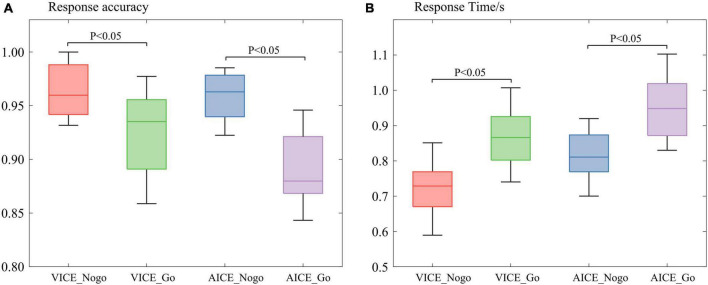
Participant’s behavioral analysis. **(A)** Participant’s average response accuracy. **(B)** Participant’s average response time.

Participants were seated comfortably, approximately 50 cm in front of a computer screen, in an electromagnetically shielded booth. At the beginning of each block, subjects were instructed by the computer program to keep track of presentations of a specific target stimulus. The visual task consisted of 6 blocks of 40 trials each (240 total trials). The auditory task consisted of 8 blocks of 30 trials each (240 total trials). Stimuli were presented for 1,500 ms and were separated by a fixation cross displayed (see Figures 3A, B). Both experiments were approved by local ethics committee.

Both cognitive experiments use the presentation to play stimuli and record participants’ behavioral data. Both cognitive experiments are conducted in a shielding room configured in our laboratory. The sound stimuli were presented to the participants via Sony MDR-7506 headphones. EEG was continuously recorded with a bandpass filter from 0.05 to 150 Hz at a sampling rate of 1,000 Hz by NeuroScan SynAmps 2 Amplifier. An ElectroCap with 64 Ag/AgCl electrodes was used to record EEG from active scalp sites referred to the tip of the nose (international 10/20 system of electrode placement). Vertical Electrooculography (VEOG) and Horizontal Electrooculography (HEOG) were recorded with two electrodes, one placed above and below the right eye and the other 10 mm from the lateral canthi. All electrode impedance was less than 5 kΩ throughout the experiment.

### 2.2 Data preprocessing

Standard EEG pre-processing techniques can clean out mixed noise, such as electrooculograms and electromyograms. The main pre-processing steps are

1. Obvious noises were removed through manual preview.
2. Electroencephalogram signals were down-sampled to 250 Hz and re-referenced to the average reference.
3. Electroencephalogram signals were filtered with a band-pass filter (FIR, 1–45 Hz).
4. Independent Component Analysis (ICA) was used to remove artifact signals such as electrooculograms and electromyograms.
5. After pre-processing, EEG signals were segmented. The moment of stimulus presentation was used as the event code in VICE. Then, we extracted the segment of EEG surrounding each stimulus (from 200 ms before a stimulus until 800 ms after the stimulus). The 200 ms period before the event code provides a baseline period. The EEG signals of each participant were divided into 240 segments. The event code in AICE was the moment of the second Chinese pronunciation. Other parameters were the same as VICE. EEG fragments with incorrect responses or prolonged response time were discarded and not included in subsequent IRP analysis.

This study divided 64 electrodes into 8 brain regions (Bian et al., 2014; Sun et al., 2019) [including: left frontal region (FL), right frontal region (FR), left temporal region (TL), right temporal region (TR), and left parietal region (PL), Right parietal region (PR), left occipital region (OL), and right occipital region (OR)]. And average the network indicators, ERPs and IRPs of all electrodes contained in each brain region.

### 2.3 Information related potentials

In this section, we focus on the EEG/ERP representation of the brain during the information processing stage. First, we briefly introduce the calculation of ERPs.

#### 2.3.1 ERP calculation theory

We assume that there are *N* trials corresponding to *N* segments of EEG in the experiment. *EEG*_*n*_ (*t*) denotes the value of the nth EEG data at moment *t (1 ≤ t ≤ T*). Thus, each segment of EEG signal can be expressed by Equation 1.


(1) $$\begin{array}{c} \begin{array}{cc} E\,E\,G_{n}\,\left(t\right)=E\,E\,G_{s_{n}}\,\left(t\right)+E\,E\,G_{e_{n}}\,\left(t\right)+E\,\left(t\right) & \\ 1\leq t\leq T,1\leq n\leq N & \end{array} \end{array}$$

Among them, *EEG*_*s_n_*_ (*t*) represents the spontaneous potential, *EEG*_*e_n_*_ (*t*) represents the evoked potential, and *E*(*t*) represents the random noise, which is approximately eliminated after data pre-processing.

It is clear from numerous studies that evoked potentials have constant waveform and constant latency. This means evoked potentials induced by the same stimulus are similar. Hence, evoked potentials can be enhanced after averaging. Spontaneous EEG is highly random and is greatly weakened after averaging. Equations 2 and 3 can express this attribute.


(2) $$\begin{array}{c} \underset{N\to \infty }{l\,i\,m}\frac{1}{N}\,\sum_{n=1}^{N}E\,E\,G_{s_{n}}\,\left(t\right)=0,1\leq t\leq T \end{array}$$

(3) $$\begin{array}{c} \underset{N\to \infty }{l\,i\,m}\frac{1}{N}\,\sum_{n=1}^{N}E\,E\,G_{e_{n}}\,\left(t\right)=E\,R\,P\,\left(t\right),1\leq t\leq T \end{array}$$

When *N* is large enough, we can calculate the ERP by Equation 4. In consequence, an important method currently to research brain cognitive activity is to calculate and compare ERPs evoked by different stimuli.


(4) $$\begin{array}{c} \begin{array}{c} E\,R\,P\,\left(t\right)\approx \frac{1}{N}\,\sum_{n=1}^{N}E\,E\,G_{n}\,\left(t\right)=\\ \frac{1}{N}\,\left(\sum_{n=1}^{N}E\,E\,G_{s_{n}}\,\left(t\right)+\sum_{n=1}^{N}E\,E\,G_{e_{n}}\,\left(t\right)\right)\\ \approx \frac{1}{N}\,\sum_{n=1}^{N}E\,E\,G_{e_{n}}\,\left(t\right),1\leq t\leq T \end{array} \end{array}$$

#### 2.3.2 IRP calculation theory

According to the ERP principle, we know that evoked potentials induced by the same stimulus are strictly time-locked and phase-locked. As a result, the ERP component is relatively stable. To ensure consistency with ERP, the same calculation method was used to analyze EEG containing information processing.

According to the ERP principle, we know that evoked potentials induced by the same stimulus are strictly time-locked and phase-locked. As a result, the ERP component is relatively stable. To ensure consistency with ERP, the same calculation method was used to analyze EEG containing information processing.

Based on the three-stage model, it is known that evoked potentials are composed of the potentials evoked by the physical, elemental, and informational attributes, denoted as *ERP*_*p*_(*t*), *ERP*_*e*_(*t*), and *ERP*_*i*_ (*t*).

If the stimulus does not contain informational attributes, its corresponding evoked potentials consist of potentials evoked by physical and elemental attributes, denoted as *ERP*_*nogo*_ (*t*) can be represented by Equation 5.


(5) E⁢R⁢Pn⁢o⁢g⁢o⁢(t)=E⁢R⁢Pn⁢o⁢g⁢op⁢(t)+E⁢R⁢Pn⁢o⁢g⁢oe⁢(t), 1≤t≤T

The Go stimulus contains three attributes, and its corresponding evoked potentials are noted as *ERP*_*go*_ (*t*) can be represented by Equation 6.


(6) E⁢R⁢Pg⁢o⁢(t)=E⁢R⁢Pg⁢op⁢(t)+E⁢R⁢Pg⁢oe⁢(t)+E⁢R⁢Pg⁢oi⁢(t), 1≤t≤T

In our experiments, the Go stimulus and Nogo stimulus have the same physical and elemental attributes. Hence, the potentials evoked by physical and elemental attributes are similar. We expressed uniformly as *ERP*_*p*_ (*t*) and *ERP*_*e*_ (*t*). *ERP*_*nogo*_ (*t*) and *ERP*_*go*_ (*t*) can be simplified to Equations 7, 8.


(7) $$\begin{array}{c} E\,R\,P_{n\,o\,g\,o}\,\left(t\right)=E\,R\,P_{p}\,\left(t\right)+E\,R\,P_{e}\,\left(t\right),1\leq t\leq T \end{array}$$

(8) E⁢R⁢Pg⁢o⁢(t)=E⁢R⁢Pp⁢(t)+E⁢R⁢Pe⁢(t)+E⁢R⁢Pg⁢oi⁢(t), 1≤t≤T

*ERP*_*go_i_*_ (*t*) is the information-related potential (IRP). In conclusion, the calculation of IRPs is


(9) I⁢R⁢P⁢(t)=E⁢R⁢Pg⁢oi⁢(t)=E⁢R⁢Pg⁢o⁢(t)-E⁢R⁢Pn⁢o⁢g⁢o⁢(t)=1N⁢∑n=1NE⁢E⁢Gg⁢on⁢(t)-1M⁢∑m=1ME⁢E⁢Gn⁢o⁢g⁢om⁢(t), 1≤t≤T

where *N* represents the number of stimuli with information and *M* represents the number of stimuli without information. In this article, *N = M*.

At this point, by constructing stimuli that only differ in information attributes, we can obtain information related potentials through Equation 9. This enables a more in-depth analysis of the cognitive components of the brain during the information processing stage.

### 2.4 Decoding model based on CSSP

It is known that EEG is composed of two parts: spontaneous and evoked potentials. In the ERP technique, the brain’s activity pattern is observed by averaging the EEG data evoked by the stimuli. To further demonstrate the specificity and significance of the brain information processing, the CSSP and machine learning methods were proposed to decoding single-trial EEG based on IRPs.

#### 2.4.1 Common spatial patterns

The common spatial patterns (CSP) algorithm is a feature extraction method that uses spatial filters to maximize the discriminability of two classes (Liang et al., 2021). CSP utilizes the diagonalization of matrices to find spatial filters that lead to new time series whose variances are optimal for the discrimination of two classes. The basic calculation principle of CSP is shown in Supplementary Material 1.

#### 2.4.2 Common spatial spectral pattern

Since different frequency bands contain different features, separating EEG into specific frequency bands can effectively eliminate redundant information. Based on what is mentioned above, the optimized spatial filter is implemented in the CSSP by inserting a time delay τ (Norizadeh Cherloo et al., 2021).

The feature extraction of CSSP is shown in Supplementary Material 2.

#### 2.4.3 Experimental parameters

In order to decode the temporal dynamics of cognitive processing in the brain, we utilized overlapping sliding windows to segment sub-epochs from each EEG recording. The window lengths were set at 200 ms, with a window shift of 100 ms. Ultimately, for each timescale *t*, the dataset comprised 240 samples (40 trials × 6 blocks in VICE or 30 trials × 8 blocks in AICE), and the duration of each sample was 200 ms ([*t*, *t* + 200]). The CSSP filter and Support Vector Machine classifier were independently trained for each participant. A fivefold cross-validation approach was employed to partition the training and test sets. The experimental flow chart was shown in Supplementary Figure 1.

## 3 Results

### 3.1 Behavioral analysis

The behavioral data were mainly concerned with the participant’s response time and response accuracy to the stimuli. The paired *t*-test was used to analyze participant’s behavior when they processed different stimuli. The results are shown in Figure 3.

In VICE, the mean response time was 0.69 s for the Nogo stimulus, which was lower than the Go stimulus (0.91 s). The paired *t*-test for response time was *p* = 0.01 < 0.05 (Cohen’s *d* = 0.97), and there was a significant difference in response time between the stimuli. Participants spent more time processing the Go stimulus than the Nogo stimulus. The mean response accuracy was 98.23% for the Nogo stimuli, which was higher than the Go stimulus (94.56%, *p* = 0.03 < 0.05, Cohen’s *d* = 1.16).

In AICE, mean response time was 1.29 s for the Nogo stimulus and 1.57 s for the Go stimulus. This difference approached significance using a paired *t*-test, *p* < 0.001 (Cohen’s *d* = 1.10). In line with VICE, participants spent more time processing the Go stimulus than the Nogo stimulus. The mean response accuracy was 99.16% for the Nogo stimulus, which was higher than the Go stimulus (93.33%). The paired *t*-test for response time was *p* < 0.001 (Cohen’s *d* = 0.82). The difference was significant.

Results of behavioral analysis illustrated that participants spend more time and get lower accuracy while processing stimuli with information. As mentioned earlier, the difference between Go and Nogo stimuli was mainly reflected in the informational attributes. The longer response time indicated that the brain spent extra time on informational attributes. Hence, the brain’s processing of stimuli included information processing stage in our experiment. The lower accuracy indicated that the brain’s processing of the informational attributes is more complex than other attributes. The results confirmed that experiments were reliable and scientific.

### 3.2 Information-related potentials

#### 3.2.1 Visual IRPs

**FIGURE 4:**
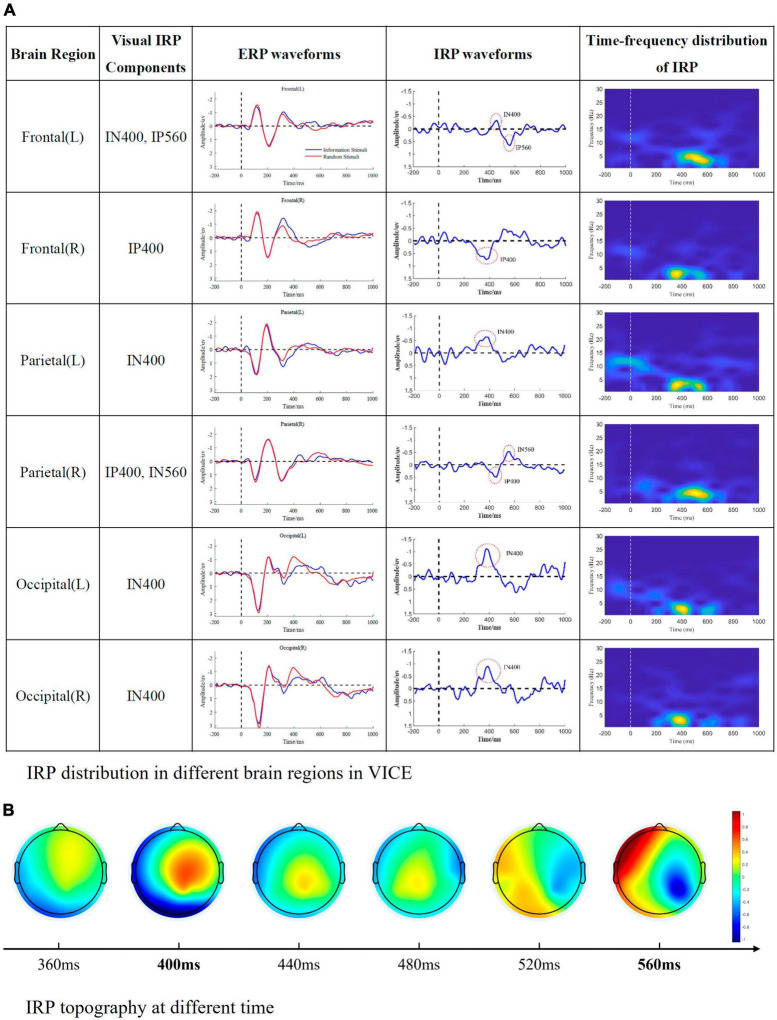
Visual IRP components. **(A)** IRP distribution in different brain regions in VICE. The first column represents the brain region, the second column represents the IRP components detected in the corresponding brain region, the third column is the ERP waveform, the fourth column is the IRP waveform, and the fifth column is the time-frequency map of IRP. **(B)** The time-varying brain topographic map of visual IRP.

To represent the connection and distinction between ERP and IRP, IP and IN were defined as positive and negative waves of the IRP components in the following work. This part mainly indicated the components observed in the visual experiment. The nature of each component and its cognitive-psychological significance will be analyzed specifically in section “4 Discussion.” The results were shown as Figure 4.

From the Figure 4, we can summarize the following conclusions.

1. Visual IRPs are mainly distributed in frontal, parietal, and occipital regions, with the main appearing moments being 400 and 560 ms.
2. There are differences in the latency of visual IRP components in different brain regions. We speculate that this might be due to the transmission of information between brain regions.
3. The main frequency distribution of visual IRP is between 1 and 6 Hz. The main appearance moments are 400 and 600 ms, which is consistent with the time domain analysis.
4. The frequency distribution of the visual IRP is obvious. Nevertheless, its energy is very low and easily masked by background noise and potentials evoked by physical and elemental attributes.

#### 3.2.2 Auditory IRPs

**FIGURE 5:**
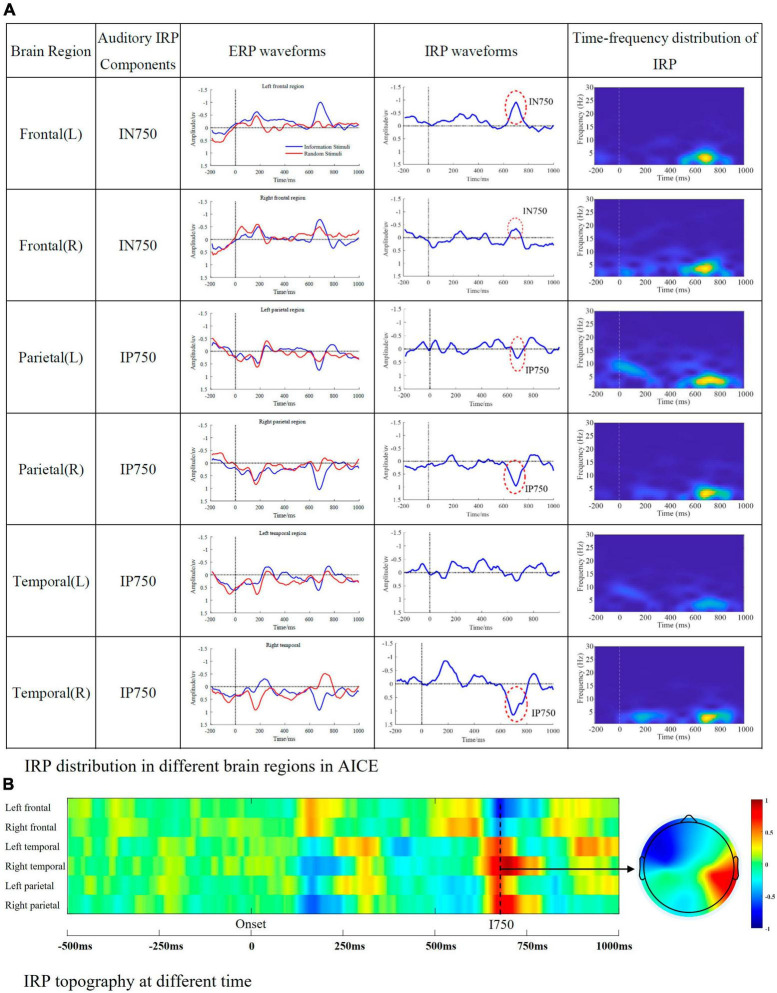
Auditory IRP components. **(A)** IRP distribution in different brain regions in AICE. The first column represents the brain region, the second column represents the IRP components detected in the corresponding brain region, the third column is the ERP waveform, the fourth column is the IRP waveform, and the fifth column is the time-frequency map of IRP. **(B)** The amplitude of auditory IRPs in different regions and brain topographic map of I750.

This part mainly indicated the components observed in the auditory experiment. The results were shown as Figure 5.

From the Figure 5, we can summarize the following conclusions.

1. Auditory IRP are mainly distributed in frontal, parietal, and temporal regions, with the main appearing moments being 750 ms.
2. The main frequency distribution of auditory IRP is between 1 and 6 Hz. The main appearance moments are between 600 and 800 ms, which is consistent with the time domain analysis.
3. The energy of auditory IRPs is low as well as visual IRPs. They are easily masked by background noise and evoked potentials caused by physical and elemental attributes.

### 3.3 Brain network analysis

In this article, the effective brain networks for visual information processing and auditory information processing were constructed using the partial directed coherence.

The key focus of this study is on the information processing stage in the cognitive processing. Based on the previous research on IRP, we constructed a visual information processing brain network by utilizing EEG signals within the range of 350–600 ms during visual experiments. Concurrently, an auditory information processing brain network was formulated employing EEG signals spanning 650–900 ms in auditory experiments. Employing paired *t*-tests, we compared the difference in node attributes between the two cognitive processes (Go and NoGo). The detailed results were presented in Table 1. Here, our study focuses on the connectivity and information processing efficiency between different brain regions, and clustering coefficients are generally considered as indicators of information processing efficiency in local brain regions of the brain network. So, we choose clustering coefficients as evaluation indicators for subsequent analysis. The research results clearly demonstrate that in VICE, the clustering coefficients exhibit a significant enhancement during the information processing stage occurring between 350 and 600 ms following the initiation of the stimulus. This suggests that, in the course of visual information processing, there is an augmented network connectivity between the frontal, parietal, and occipital regions of the brain, accompanied by closer local brain network connections.

**TABLE 1:** Differences in clustering coefficients between two cognitive processes.

	VICE				AICE			
	**Go**	**NoGo**	***p***	**Cohen’s *d***	**Go**	**NoGo**	***p***	**Cohen’s *d***
FL	0.19 ±0.02	0.17 ±0.03	**0.04**	0.78	0.22 ±0.03	0.20 ±0.02	**0.01**	0.78
FR	0.22 ±0.01	0.19 ±0.03	**0.01**	1.34	0.25 ±0.03	0.22 ±0.03	**<0.01**	1.00
PL	0.22 ±0.01	0.20 ±0.02	**0.01**	1.26	0.23 ±0.02	0.21 ±0.04	**0.04**	0.64
PR	0.18 ±0.02	0.15 ±0.03	**0.02**	1.18	0.20 ±0.03	0.16 ±0.02	**0.01**	1.57
TL	0.19 ±0.02	0.18 ±0.02	0.22	0.50	0.25 ±0.04	0.22 ±0.02	**0.02**	0.95
TR	0.20 ±0.01	0.19 ±0.02	0.25	0.63	0.24 ±0.03	0.20 ±0.03	**<0.01**	1.33
OL	0.24 ±0.02	0.22 ±0.02	**0.01**	1.00	0.18 ±0.02	0.18 ±0.01	0.71	0.01
OR	0.22 ±0.04	0.20 ±0.03	**0.03**	0.57	0.19 ±0.01	0.2 ±0.01	0.48	1.00

The bold values indicate significant difference.

Analogously, the results of AICE are similar to those of VICE, indicating a notable enhancement in the clustering coefficients of brain network nodes within the frontal, parietal, and temporal regions during the auditory information processing stage. The analytical outcomes of the brain networks align closely with the earlier research findings on IRP and CSSP.

**FIGURE 6:**
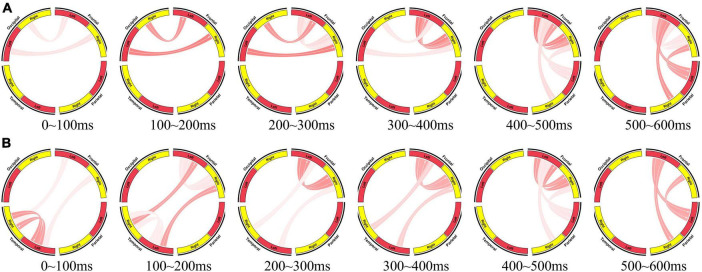
Brain network for information processing. **(A)** Brain network for visual information processing (0∼600 ms, the stimulus presentation time is taken as 0 time). **(B)** Brain network for auditory information processing (0∼600 ms, the end time of the second element is taken as 0 time).

Following this, we employed the same methodology to construct brain networks at various time points during the cognitive process, as illustrated in Figure 6. The same overlapping sliding windows was used to segment sub-epochs from each EEG recording. The window lengths were set at 200 ms, with a window shift of 100 ms. In the brain network for visual information processing, the stimulus presentation time was taken as 0 time. In the brain network for auditory information processing, the end time of the second element was taken as 0 time. This systematic approach ensured uniformity in the segmentation process and facilitated comparative analysis across different experimental conditions.

At the initial stage of visual information processing (0∼200 ms), the occipital region transmits the received visual stimuli to the frontal region, and the frontal region performs primary processing. At 200∼300 ms, initial information interaction begins in the left and right frontal areas. At 300∼400 ms, the proportion of information interaction between left and right frontal regions becomes larger, while the information transfer between occipital and frontal regions weakens. At 400∼500 ms, frontal region transfers the processing results of stimuli to parietal region. And at 500∼600 ms, the information interaction between left and right frontal regions decreases, and the information transfer from frontal region to parietal region increases, indicating that the information processing tends to end. The parietal region completes the response to the stimuli (press the mouse).

At the initial stage of auditory information processing, the left and right temporal regions receive and process auditory stimuli. Then they transmit the preliminary processing results and stimuli to the frontal region. After this, visual information processing and auditory information processing showed a high degree of consistency. The left and right frontal regions interact to further process the received auditory stimuli. Then, the information flows from the frontal region to the parietal region.

The effective brain network clearly reflects the collaboration of brain regions during brain information processing. It also reveals the consistency of visual information processing and auditory information processing, and also provides the evidence for the consistency of I400 and I750.

### 3.4 Decoding the brain’s cognitive processes

Electroencephalogram signals are event-related, and different frequency bands contain different features. In this section, we use the identification of IRP on a single trial to decode the cognitive processes in the brain. As a result, CSSP was applied to extract EEG features by separating EEG into specific frequency bands and specific brain regions. The three-dimensional features of time, frequency and space were fused by CSSP. Consequently, Support Vector Machine classifiers were trained by CSSP features to distinguish whether single-trial EEG signals contains information processing.

To enhance the decoding of EEG signals, we process EEG in the temporal, frequency, and spatial domains.

#### 3.4.1 Frequency domain

Cognitive experiment results indicated that the main frequencies of IRP were in the range of 1–6 Hz for both visual and auditory. For this reason, the EEG data were filtered off-line through a 1-Hz to 6-Hz passband phase-shift-free Butterworth filter.

#### 3.4.2 Spatial domain

According to the results, visual information processing include frontal, parietal, and occipital regions. Furthermore, brain asymmetry has been observed in the experiments. Twenty-four channels covering the above brain regions were selected for analysis and classification. For auditory information processing, the main brain regions involve frontal, parietal, and temporal regions. Brain asymmetry also was observed in auditory experiments. As a result, 22 EEG channels covering the above brain regions were selected for analysis and classification.

#### 3.4.3 Temporal domain

In terms of the IRPs, the latency between different regions was about 12 ms. The difference was consistent in auditory and visual experiments. For this reason, the time delay factor in CSSP was set to 12 ms.

**FIGURE 7:**
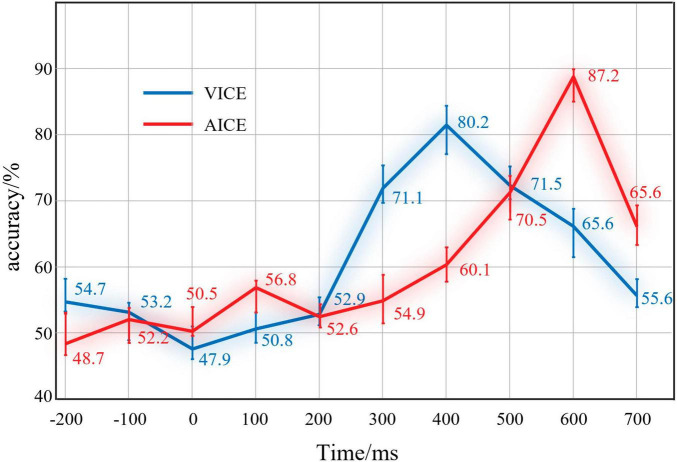
Results of IRPs classification at different periods. The red line was the results of AICE and the blue line was the results of VICE.

Accuracy was employed to evaluate the performance of classification. For each timescale, the average accuracy of all participants in this timescale was used as result. The results are shown in Figure 7.

The results of visual EEG displayed that the recognition rate was 54.7% before the stimulus. At this time, EEG signals mainly consist of spontaneous potential. This mean that there was no obvious distinction between the two kinds of EEG signals. After the stimulus, the recognition rate did not increase significantly in 0 ∼ 400 ms. In this stage, the brain is currently processing the physical and elemental attributes of the stimuli. Both Go stimuli and Nogo stimuli had the same physical and elemental attributes. In terms of ERP theory, they have stimuli evoked potentials. For this reason, the distinction is not obvious. After that, the recognition rate increased significantly in 400∼600 ms and reached 80.2%. This indicated that the difference between the two kinds of EEG reached the maximum. The brain mainly processes information attributes at this stage. The results clear support for the IRPs. Subsequently, the recognition rate decreased, which indicated that the brain completed the information processing.

The results of auditory EEG were similar to visual EEG. The only difference was that the accuracy reached 87.2% at 600∼800 ms in the auditory experiment. Auditory information processing stage is later than vision in our experiments. Pattern recognition results demonstrated that the brain could produce components with obvious recognition when processing information.

## 4 Discussion

According to the results of cognitive experiments, the brain generates a series of cognitive components (IRPs) during information processing stage. The IRPs were observed in occipital, temporal, frontal, and parietal regions. Among these brain regions, the occipital and temporal region is the receiving brain region for visual and auditory information (Bentin et al., 1993; Gămănut̨ and Shimaoka, 2022), the frontal region is the main brain region for information processing (Raggi and Ferri, 2020), and the parietal region is the responding brain region (Culham and Valyear, 2006). The input, processing and response of information are reflected in the experimental results.

### 4.1 IRP components

Figures 4, 5 list the main IRP components observed in the cognitive experiments. The IRPs were summarized according to their latency: IN400 vs. IP400, IP560 vs. IN560 in visual processing, IN750 vs. IP750 in auditory processing. They were analyzed and discussed in turn below.

#### 4.1.1 IN400 and IP400 (I400)

In VICE, around 400 ms, a significant negative wave (IN400) appeared in the left frontal, left parietal and occipital regions; a significant positive wave (IP400) appeared in the right frontal region. As mentioned in the introduction, the representative ERP component in the information processing stage is N400 in the current research. I400 and N400 have both similarities and differences. The similarities include: (1) I400 and N400 have similar latency (approximately 400 ms). (2) Current research demonstrated that non-verbal stimuli such as faces and images can also induce N400 (Jin et al., 2014; Olivares et al., 2015). Moreover, current research found that N400 is related to the extraction of semantic information and memory (Hofmann and Jacobs, 2014; Feng et al., 2019). And semantics is information’s core content. In this sense, N400 is related to the information processing. According to information theory, receiving information aims to reduce uncertainty. The fixed arrangement of elements also reduces uncertainty. Therefore, it is reasonable and reliable to establish a connection between IN400/IP400 and N400. The primary distinction lies in the identified brain regions. Typically, the N400 exhibits its maximum amplitude over central and parietal electrode sites, with a slightly greater magnitude over the right hemisphere compared to the left hemisphere (Lau et al., 2008; Wang et al., 2012; Panda et al., 2021). The second dissimilarity pertains to polarity. Existing studies have not reported a polarity reversal (positive/negative) between the left and right hemispheres. The I400 appears inconsistent with these characteristic features of the N400. Consequently, future research endeavors will delve deeper into examining the relationship between I400 and N400 to substantiate the connection between these two phenomena.

#### 4.1.2 IN560 and IP560 (I560)

In VICE, a negative wave was observed at 560 ms in the right parietal region, defined as IN560. A positive wave was observed at 560 ms in the left frontal region, defined as IP560. There is no specific study of this observation. To further understand the mechanism of I560, we mapped the time-varying brain topography of visual IRP, as shown in Figure 4A. The brain topography displayed that activation in brain regions gradually transferred from frontal regions to parietal regions from 400 to 600 ms. This indicates that there is a connection between the frontal and parietal regions. According to the current understanding of brain regions, the frontal region is responsible for information processing, and the parietal region is the motor-related brain region. Consequently, the connection between the two regions masks the end of information processing and the beginning of information response. The brain network results in Figure 6A further substantiates the above viewpoint. Specifically, during the 500–600 ms, the interaction between the left and right frontal regions weakens, which is reflected as a positive wave in the frontal region. Correspondingly, the interaction in the frontal and parietal regions is enhanced, presenting as negative waves in the parietal region. In the visual brain decoding experiment, the recognition accuracy (71.5% and 65.6%) for the time periods of 500–700 and 600–800 ms was significantly higher than the baseline level (50%). Therefore, the distinguishability of EEG in these two time periods primarily comes from I560. Subsequently, recognition accuracy decreased to a level approximating the baseline, providing an additional perspective that the brain’s information processing process has been completed at this time. In summary, we consider that I560 is a potential change that occurs when information processing in the frontal regions diminishes and transmits the results to the parietal regions, signifying the conclusion of the brain’s information processing stage.

#### 4.1.3 IN750 and IP750 (I750)

In AICE, around 750 ms, a significant negative wave (IN750) appeared in the frontal regions; a significant positive wave (IP750) appeared in the parietal and right temporal regions. There is also no specific study of this component. Compared with I400 in VICE, we considered that I750 is consistent with I400. The reasons are as follows: (1) I750 and I400 have similar energy intensity and range in the frequency spectrum. (2) The distribution of brain regions was essentially the same. (3) Brain network results suggest that visual and auditory information processing have similar processing, which is highly likely to induce similar components. The difference between I750 and I400 focuses on the latency. Since the auditory stimulus is time-series, the brain needs enough time to receive the complete information. Conversely, the brain can receive complete information in a very short time due to the particularity of vision. To further investigate the mechanism of I750, we compared the auditory IRP waveform and auditory stimulus waveform. According to Chinese grammar, semantic information is generated when the second element is fully apprehended by the brain. The average duration of the second element is about 350 ms. If the end time of the second element is used as the event code, the latency of I750 is 400 ms, which is consistent with the latency of I400. Therefore, it is highly likely that I750 is a manifestation of N400 or I400 in the processing of auditory information.

In this article, we propose the concept of IRPs and show that this is a unified EEG\ERP representation of the brain in information processing. IRP is a comprehensive record of the continuous and complex activity of the brain during information processing, and is a composite representation of many evoked potentials, fusion and interaction. IRP contains a large amount of information about the high-level components and laws related to information processing and is a set of codes that record the information cognitive laws of the human brain. The effective IRP is very weak, changes dynamically with time, and is deeply hidden in the spurious signal. The analysis of IRP must be done from multiple perspectives, such as time domain, frequency domain and space domain, to comprehensively analyze the nature and laws of IRP.

Information-related potential is an extension to ERP. At the processing level, ERP is an external representation of the physical and elemental processing stages of the brain, while IRP is an external representation of the information processing stage of the brain. On the choice of experimental paradigm and stimulus materials, ERP can take full advantage of various paradigms and stimulus materials to explore the nature of ERP, but IRP experiments require special experimental designs and more demanding stimulus materials to ensure the extractability of IRP. In IRP experiments, the experimenter-designer must fully consider the physical, elemental, and informational attributes of the stimulus material to minimize the influence of other factors while meeting the requirements of the study.

### 4.2 Limitations

Due to pioneering and exploratory characteristics of the current contribution, there are several issues which need to be considered and, on some occasions, also improved, when using the analysis method in the future.

Although we proposed IRPs, we cannot give an effective answer about how to define the information effectively and how much information the stimulus contains. We need more research to define the concept of information accurately. In addition, we will consider how to how to quantify the amount of information in terms of information theory. The quantification of information is the key to follow-up IRP research.

There are disadvantages to the experimental design, such as the unbalance of the participants. In our experiments, the unbalance of the genders and relatively small age range among participants (18–24 years) need also be acknowledged. Age-related decline in auditory temporal processing start to emerge in middle-aged adults and are suggested to occur due to changes in auditory processing in the central auditory system. Thus, the large age range of the participants may produce different IRPs. The same problem is also reflected in the proportion of genders. Consequently, we will compensate for these disadvantages in future experiments and explore IRP through more scientific and reasonable experiments.

Finally, the consistency of auditory IRP and visual IRP needs to be demonstrated by more practical experiments, which is the focus of future work. For example, we did not find an I560-like component in AICE. We speculate that there should be an I560-like component in the auditory IRP. However, due to the special syntax of the Chinese language and experimental setup, the auditory stimulus contains three elements, and the I560 component is most likely to be submerged in the subsequent auditory processing. We will verify this idea in subsequent work.

## 5 Conclusion

In this article, we introduce the concept of IRPs and posit that it serves as a unified EEG/ERP representation of the brain during information processing. We propose a methodology to elucidate how the brain manages the informational attributes of stimuli, considering that stimuli typically comprise fixed elements, and the arrangement of these elements reflects their informational attributes. Visual and auditory information-related cognitive experiments were conducted to probe into the brain’s information processing procedures, yielding a series of cognitive components associated with information processing. Then, we validated the consistent EEG patterns of audio-visual perceptual holistic through brain network and brain decoding experiments.

In forthcoming studies, we plan to conduct more precise experiments to delve into the detailed analysis of the information processing processes represented by IRP and validate the consistency between visual IRP and auditory IRP.

## Data availability statement

The raw data supporting the conclusions of this article will be made available by the authors, without undue reservation.

## Ethics statement

The studies involving humans were approved by the Heilongjiang Provincial Hospital Ethics Committee. The studies were conducted in accordance with the local legislation and institutional requirements. The participants provided their written informed consent to participate in this study.

## Author contributions

HWL: Data curation, Formal analysis, Investigation, Methodology, Software, Validation, Visualization, Writing – original draft. HFL: Conceptualization, Funding acquisition, Methodology, Project administration, Supervision, Writing – review & editing. LM: Data curation, Formal analysis, Funding acquisition, Investigation, Project administration, Supervision, Visualization, Writing – original draft, Writing – review & editing. DP: Data curation, Formal analysis, Investigation, Software, Writing – original draft.
