# Supplementary material for: Revealing brain’s cognitive process deeply: a study of the consistent EEG patterns of audio-visual perceptual holistic
Source: Front Hum Neurosci. 2024 Mar 27;18:1377233. doi: 10.3389/fnhum.2024.1377233 (PMC11004307; doi:10.3389/fnhum.2024.1377233)
Supplement: Supplementary file 1 [file Image_1.PDF]

# Supplementary Material

## 1 Common Spatial Patterns

The common spatial patterns (CSP) algorithm is a feature extraction method that uses spatial filters to maximize the discriminability of two classes. CSP utilizes the diagonalization of matrices to find spatial filters that lead to new time series whose variances are optimal for the discrimination of two classes.

The basic calculation principle of CSP is as follows. Given 2 sets of signal samples, noted class $\mathbb{X} = \{X_1, X_2, \dots X_n, \dots X_N\}$ and class Y= $\{Y_1, Y_2, \dots Y_M\}$, $X_n, Y_m \in \mathbb{R}^{dc\times dt}$.

(1) Calculate the average covariance matrix for each class.

$$\Sigma_X = \frac{1}{N}\sum_{n=1}^{N} \frac{X_n X_n^T}{trace(X_n X_n^T)} \tag{10}$$

$$\Sigma_Y = \frac{1}{M}\sum_{m=1}^{M} \frac{Y_m Y_m^T}{trace(Y_m Y_m^T)} \tag{11}$$

Among them, $\Sigma_X$ and $\Sigma_Y$ denotes the average covariance matrix of the set $\mathbb{X}$ and $\mathbb{Y}$. Trace (.) is function which calculates sum of diagonal of matrix.

(2) Calculate $\Sigma_{XY}$ which denotes the sum of the average covariance matrix of the two classes.

$$\Sigma_{XY} = \Sigma_X + \Sigma_Y \tag{12}$$

(3) Decompose the covariance matrix obtained in (3).

$$\Sigma_{XY} = U\Lambda U^T \tag{13}$$

$\Lambda$ denotes the diagonal matrix constructed from the eigenvalues in descending order and **U** is the matrix of eigenvectors corresponding to the eigenvalues.

(4) The whitening transformation matrix **P** is constructed using the eigenvector matrix and the diagonal array of eigenvalues.

$$P = \frac{U^T}{\sqrt{\Lambda}} \tag{14}$$

(5) $\Sigma_X$ and $\Sigma_Y$ are then transformed as

$$\begin{aligned} S_X &= P\Sigma_X P^T \\ S_Y &= P\Sigma_Y P^T \end{aligned} \tag{15}$$

(6) Decompose $S_X$ and $S_Y$ by eigenvalue.

$$S_X = U_X \Lambda_X U_X^T$$
$$S_Y = U_Y \Lambda_Y U_Y^T \quad (16)$$

$\Lambda_x$ and $\Lambda_Y$ denote the diagonal matrix constructed from the eigenvalues of $S_X$ and $S_Y$. **$U_X$** and **$U_Y$** are the eigenvector matrix corresponding to the eigenvalues.

It can be proved that $\Lambda_x$, $\Lambda_Y$, **$U_X$** and **$U_Y$** have the following attributes

$$U_X = U_Y$$
$$\Lambda_X + \Lambda_Y = I \quad (17)$$

where ***I*** represent the identity matrix.

We can see that $\Lambda_x$ and $\Lambda_Y$ are negatively correlated. More precisely, when the eigenvalue of $S_X$ is the largest, the eigenvalue of $S_Y$ is the smallest. Sort the $S_X$'s feature values and feature vectors in descending order.

(7) Construct the spatial filter **W**.

$$W = U_X^T P = U_Y^T P \quad (18)$$

The filter **W** is constructed by the feature vector and the whitening matrix. According to the above attributes, the filter W concentrates the features of $\mathbb{X}$ to the head and the features of $\mathbb{Y}$ to the tail.

For the unknown sample $Q \in \mathbb{R}^{dc \times dt}$, the CSP features are extracted by the following equation.

$$Z = W \times Q \quad (19)$$

## 2 Common Spatial Spectral Pattern

Since different frequency bands contain different features, separating EEG into specific frequency bands can effectively eliminate redundant information. Based on what's mentioned above, the optimized spatial filter is implemented in the common spatial spectral pattern (CSSP) by inserting a time delay τ.

The feature extraction of CSSP is

$$Z = W_0 \times Q + W_\tau \times Q_\tau \quad (20)$$

where $Q_\tau$ represents the sample after a time delay τ, $W_0$ represents the spatial filter of the original sample, and $W_\tau$ represents the spatial filter of the sample after a time delay.

At this point, the optimization objective is to solve for $W_0$ and $W_\delta$. To solve the above optimization problem, we append the delayed vectors $Q_\tau$ as additional channels to $Q$, i.e.,

$$\widehat{X_n} = \begin{pmatrix} X_n \\ X_{n\tau} \end{pmatrix}, 1 \le n \le N \quad (21)$$

$$\widehat{Y_n} = \begin{pmatrix} Y_m \\ Y_{m\tau} \end{pmatrix}, 1 \le m \le M \tag{22}$$

where $X_{n\tau}$ and $Y_{n\tau}$ are the new sample after appending. At this point, the CSSP problem is converted into a CSP problem, and the spatial filter $\widehat{W}$ is calculated using the calculation method in 2.3.1. $\widehat{W}$ is composed of $W_0$ and $W_\delta$.

$$\widehat{W} = \begin{pmatrix} W_0 \\ W_\tau \end{pmatrix} \tag{23}$$

For the unknown sample $Q \in \mathbb{R}^{dc \times dt}$, the CSSP features can be extracted by the following equation.

$$Z = \widehat{W} \times \begin{pmatrix} Q \\ Q_\tau \end{pmatrix} \tag{24}$$

The signal passing the filter $\widehat{W}$ is equivalent to the original signal's projection of each line vector. We use the variance of each row in ***Z*** as the final feature vector. Use $z_i$ represent the *i*-th row of ***Z*** (*i*= *1,2, ..., NC*). The variance of this row can be defined as follows.

$$v_i = \ln\left(\frac{var(z_i)}{\sum_{j=1}^{dc} var(z_j)}\right) \tag{25}$$

All row vectors' variance of ***Z*** is combined as its CSSP feature called ***F***. then, ***F*** is sent to the classifier for classification.

$$F = [v_1, v_2, \dots, v_{dc}]^T \tag{26}$$

## 3 Supplementary Figures and Tables

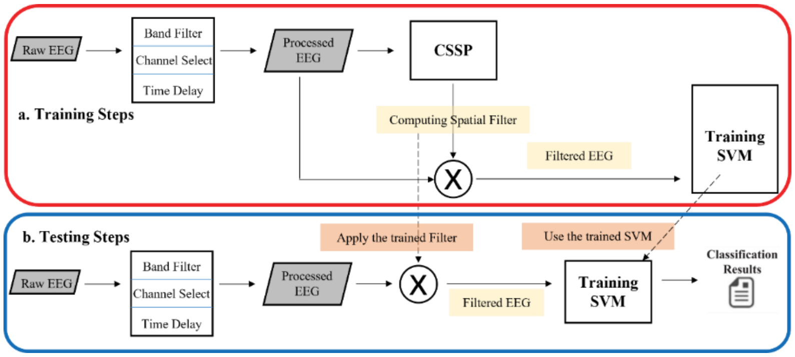


**Fig. S1. Details of the training and testing in our experiments.** (**A**) Training Steps. The train dataset was used to train Spatial Filter and SVM Classifier. (**B**) Testing Steps. EEG features was extract by Spatial Filter and sent to SVM Classifier.
